# Specific changes and clinical significance of plasma D-dimer during pregnancy and puerperium: a prospective study

**DOI:** 10.1186/s12884-023-05561-1

**Published:** 2023-04-13

**Authors:** Qin Xu, Li Dai, Hong-Qin Chen, Wei Xia, Qi-Lin Wang, Cai-Rong Zhu, Rong Zhou

**Affiliations:** 1 Department of Obstetrics and Gynecology, Center for Translational Medicine, Key Laboratory of Birth Defects and Related Diseases of Women and Children (Sichuan University), Ministry of Education, West China Second University Hospital, Sichuan University; NHC Key Laboratory of Chronobiology, Sichuan University, Chengdu, 610041 China; 2 Department of Epidemiology and Health Statistics, West China School of Public Health and West China Fourth Hospital, Sichuan University, Chengdu, 610041 China

**Keywords:** Plasma D-dimer, Pregnancy, Puerperium, Reference range, Venous thromboembolism

## Abstract

**Background:**

Pregnant and puerperal women are high-risk populations for developing venous thromboembolism (VTE). Plasma D-dimer (D-D) is of good value in the diagnosis of exclusion of VTE in the nonpregnant population. Since there is no consensus reference range of plasma D-D applicable to pregnant and puerperal women, the application of plasma D-D is limited. To investigate the change characteristics and the reference range of plasma D-D levels during pregnancy and puerperium and to explore the pregnancy- and childbirth-related factors affecting plasma D-D levels and the diagnostic efficacy of plasma D-D for excluding VTE during early puerperium after caesarean section.

**Methods:**

A prospective cohort study was conducted with 514 pregnant and puerperal women (cohort 1), and 29 puerperal women developed VTE 24–48 h after caesarean section (cohort 2). In cohort 1, the effects of the pregnancy- and childbirth-related factors on the plasma D-D levels were analyzed by comparing the differences in plasma D-D levels between different groups and between different subgroups. The 95th percentiles were calculated to establish the unilateral upper limits of the plasma D-D levels. The plasma D-D levels at 24–48 h postpartum were compared between normal singleton pregnant and puerperal women in cohort 2 and women from the cesarean section subgroup in cohort 1, binary logistic analysis was used to analyze the relevance between plasma D-D level and the risk of VTE developing 24–48 h after caesarean section, and a receiver operating characteristic (ROC) curve was used to assess the diagnostic efficacy of plasma D-D for excluding VTE during early puerperium after caesarean section.

**Results:**

The 95% reference ranges of plasma D-D levels in the normal singleton pregnancy group were ≤ 1.01 mg/L in the first trimester, ≤ 3.17 mg/L in the second trimester, ≤ 5.35 mg/L in the third trimester, ≤ 5.47 mg/L at 24–48 h postpartum, and ≤ 0.66 mg/L at 42 days postpartum. The plasma D-D levels of the normal twin pregnancy group were significantly higher than those of the normal singleton pregnancy group during pregnancy (P < 0.05), the plasma D-D levels of the GDM group in the third trimester were significantly higher than those of the normal singleton pregnancy group (P < 0.05). The plasma D-D levels of the advanced age subgroup at 24–48 h postpartum were significantly higher than those of the nonadvanced age subgroup (P < 0.05), and the plasma D-D levels of the caesarean section subgroup at 24–48 h postpartum were significantly higher than those of the vaginal delivery subgroup (P < 0.05). The plasma D-D level was significantly correlated with the risk of VTE developing at 24–48 h after caesarean section (OR = 2.252, 95% CI: 1.611–3.149). The optimal cut-off value of plasma D-D for the diagnosis of exclusion of VTE during early puerperium after caesarean section was 3.24 mg/L. The negative predictive value for the diagnosis of exclusion of VTE was 96.1%, and the area under the curve (AUC) was 0.816, P < 0.001.

**Conclusions:**

The thresholds of plasma D-D levels in normal singleton pregnancy and parturient women were higher than those of nonpregnant women. Plasma D-D had good value in the diagnosis of exclusion of VTE occurring during early puerperium after caesarean section. Further studies are needed to validate these reference ranges and assess the effects of pregnancy- and childbirth-related factors on plasma D-D levels and the diagnostic efficacy of plasma D-D for excluding VTE during pregnancy and puerperium.

## Background

A series of unique physiological and anatomical changes occur during pregnancy and puerperium. To adapt to the process of pregnancy and childbirth, maintain the integrity of the placenta and reduce postpartum hemorrhage, the coagulation system is highly activated, and the fibrinolytic system is secondarily activated. These two systems maintain a new dynamic balance so that the blood is in a physiological hypercoagulable state during pregnancy [1, 2]. The hypercoagulable state of the blood continues to be maintained in the early puerperium, and then it returns to the pregnancy level around 2–4 weeks postpartum [3]. Women simultaneously possess the Virchow triad of altered coagulation, stasis, and vascular damage during pregnancy and puerperium [4], placing them at high risk for venous thromboembolism (VTE). Their incidence and mortality from VTE are higher than those of the nonpregnant healthy population. Compared with nonpregnant women, the risk of VTE has been reported to be approximately five-fold higher during pregnancy, while the risk of VTE is further increased up to more than 20-fold during puerperium [5]. VTE is a serious perinatal complication that can lead to severe adverse maternal outcomes and can even be life-threatening. Currently, the diagnostic approach to VTE in the nonpregnant population is based on a combination of clinical scoring systems, blood tests, and imaging using compression ultrasound, ventilation-perfusion scans, or computed tomography pulmonary angiography [6]. A maternal clinical scoring system has not been clinically validated, and the clinical manifestations of VTE are occult and nonspecific. Ventilation-perfusion scanning and computed tomography pulmonary angiography have certain radiation effects on the fetus and maternal breast [7]. Therefore, blood markers that can assist in early identification and facilitate dynamic monitoring of maternal coagulation and fibrinolysis status are needed in clinical practice.

D-dimer (D-D) is a relatively stable cross-linked fibrin degradation product. It is an important blood indicator of a high blood coagulation status and reflects the presence of thrombosis and secondary fibrinolytic hyperfunction [8]. At present, plasma D-D is mainly used to assist in the exclusion of a diagnosis of VTE [9, 10]. The latest meta-analysis concluded that the negative predictive value of plasma D-D to exclude the occurrence of acute VTE in pregnant women was nearly 100% (95% CI: 99.19–100.0%), suggesting that plasma D-D can effectively exclude the occurrence of VTE in low-moderate risk pregnant women, but the analysis emphasized the poor diagnostic specificity of plasma D-D [11]. Since most studies have found that the plasma D-D levels of pregnant and puerperal women are generally higher than those of nonpregnant women, it is considered that the reference range of plasma D-D levels for screening VTE in nonpregnant women (≤ 0.5 mg/L) is not suitable for pregnant and puerperal women, which limits the application of plasma D-D. In addition, the vast majority of current guidelines consider multiple adverse pregnancy- and childbirth-related factors such as multiple pregnancy, preeclampsia, advanced age, obesity, ovarian hyperstimulation syndrome (OHSS) induced by assisted reproduction, and caesarean section as risk factors for VTE [12–16]. Most researchers believe that gestational diabetes mellitus (GDM) can also affect coagulation function and increase the incidence of VTE by damaging the vascular endothelium [17]. A case-control study found that GDM was associated with an increased risk of maternal VTE (OR = 4.95% CI: 2.0-8.9) [18].

Based on the status quo, our study aimed to determine the change characteristics of plasma D-D levels during pregnancy and puerperium; establish a reference range of plasma D-D levels for pregnant and puerperal women; investigate the effects of pregnancy and childbirth-related factors, including multiple pregnancies, hypertensive disorders of pregnancy (HDP), GDM, advanced age, obesity, conception mode and delivery mode, on plasma D-D levels; and assess the diagnostic efficacy of plasma D-D on VTE during early puerperium after caesarean section.

## Methods

### Patient selection and data extraction

After an extremely rigorous estimation of the required sample size, a prospective cohort study was conducted to recruit pregnant and puerperal women who received regular prenatal care throughout pregnancy, delivered and had 42-day postpartum follow-up at West China Second University Hospital from March 2020 to February 2022 as cohort (1) And there are some women who received regular prenatal care in other hospitals but delivered and had 42-day postpartum follow-up at West China Second University Hospital during the corresponding period, we recruited these women who developed VTE within 24–48 h after caesarean section as cohort (2) The age of both cohorts was 18–45 years old, and we excluded women who met the following criteria: (1) missed 42-day postpartum follow-up and had incomplete clinical data; (2) had cardiovascular disease, liver or kidney disease, thrombotic disease, infectious disease, immune disease, malignant tumor or other underlying diseases; (3) had a history of spontaneous abortion, stillbirth or smoking; (4) received drugs affecting coagulation and fibrinolysis during the perinatal period; or (5) had fetal malformations or stillbirth in this pregnancy.

Through the electronic medical record system of our hospital, the relevant data of all subjects were collected, including the basic maternal characteristics of this pregnancy (height, pre-pregnancy weight, previous number of deliveries, conception mode, single/twin pregnancy, delivery age, gestational week of delivery and delivery mode) and maternal outcome indicators: HDP, GDM, VTE, and postpartum hemorrhage (PPH). The gestational week was verified through fetal growth parameters measured by ultrasonography in the first trimester. According to the regulations of the International Federation of Gynecology and Obstetrics (FIGO), advanced maternal age (AMA) is defined as a woman’s age at childbirth of ≥ 35 years old. According to the relevant standards published by the China Obesity Working Group in 2003, obesity is defined as a body mass index (BMI) ≥ 28.0 kg/m^2^. We diagnosed subjects with HDP according to the practice guidelines issued by the International Society of Hypertension (ISH) in 2020 [19]. GDM was diagnosed according to the diagnosis and treatment guidelines issued by the American Diabetes Association (ADA) in 2020 [20].

Cohort 1 was divided into a normal singleton pregnancy group, a normal twin pregnancy group, an HDP group and a GDM group. The normal singleton pregnancy group was divided into advanced age (≥ 35 years) and nonadvanced age (< 35 years) subgroups according to delivery age, obese (BMI ≥ 28 kg/m^2^) and nonobese (BMI < 28 kg/m^2^) subgroups according to pre-pregnancy BMI, natural fertilization and in vitro fertilization-embryo transplantation (IVF-ET) subgroups according to conception mode, or caesarean section and vaginal delivery subgroups according to delivery mode. We collected blood samples from cohort 1 at three different pregnancy stages (first trimester: 10–13^+ 6^ weeks of gestation, second trimester: 20–27^+ 6^ weeks of gestation and third trimester: 32–40^+ 6^ weeks of gestation) and at two postpartum stages (24–48 h postpartum and 42 days postpartum). The blood samples of cohort 2 were collected at 24–48 h postpartum.

### Laboratory testing

Peripheral venous blood was collected in an anticoagulant tube containing 0.2 ml (10^9^ mmol/L) sodium citrate solution, mixed immediately and centrifuged at 3000 r/min for 15 min. The D-D concentration was measured with a quantitative latex microparticle enhanced turbidimetric immunoassay on an OPBP07IFU2015A (Siemens Company in Germany) automated coagulation analyzer. Plasma was analyzed within 2 h of collection. D-D concentrations (mg/L) were expressed in fibrinogen-equivalent units (FEU). These tests were performed according to the standard operating procedures (SOPs) of the instrument.

### Statistical analyses

Data analyses were performed using the software package SPSS statistics version 26.0 (SPSS Inc.). Data that conformed to the normal distribution are expressed as the mean ± standard deviation (${\rm{\bar X}} \pm {\rm{S}}$), and then the homogeneity of variance was tested. If the variance was equal, one-way ANOVA was used to analyze the difference between the two groups; if the variance was unequal, the rank-sum test was used. Data that were not normally distributed are expressed as the median and interquartile range [M (P25, P75)], and the Mann‒Whitney U test was used to compare the differences between groups. The enumeration data are expressed as a percentage (%), and the differences between the two groups were compared using the chi-square test. In cohort 1, MANOVA of repeated measurements was used to analyze the difference in plasma D-D levels in normal singleton pregnancies at 5 periods. A linear mixed-effects model was used to analyze the relationship between plasma D-D levels and gestational week. According to the data characteristics of our study, the method proposed by Jennen-Steinmetz was selected to estimate the sample size for the reference range [21]. To establish a unilateral reference range of the nonnormal distribution data, the calculation formula we used was n = q(1 − q) (Z_(1+η)/2_/δ)^2^. n refers to the required sample size; Z_(1+η)/2_ refers to the critical value corresponding to a normal distribution; q refers to the percentile of the reference range; *δ* refers to the allowable error. Combined with the actual research needs, q was 0.95, *η* was not less than 0.8, *δ* was 0.015, and the sample size was calculated to be at least 347 cases. The 95% reference interval of plasma D-D was calculated by the unilateral upper limit. The plasma D-D levels at 24–48 h postpartum were compared between normal singleton pregnant and puerperal women in cohort 2 and women from the cesarean section subgroup in cohort 1, binary logistic analysis was used to analyze the relevance of the correlation between the plasma D-D level and the risk of VTE developing 24–48 h after caesarean section, and a receiver operating characteristic (ROC) curve was used to calculate the optimal cut-off value of the plasma D-D levels for the diagnosis of VTE occurring during early puerperium after caesarean section and to assess the diagnostic efficacy. A P value < 0.05 for both sides was considered significant.

## Results

### Study population

**Fig. 1:**
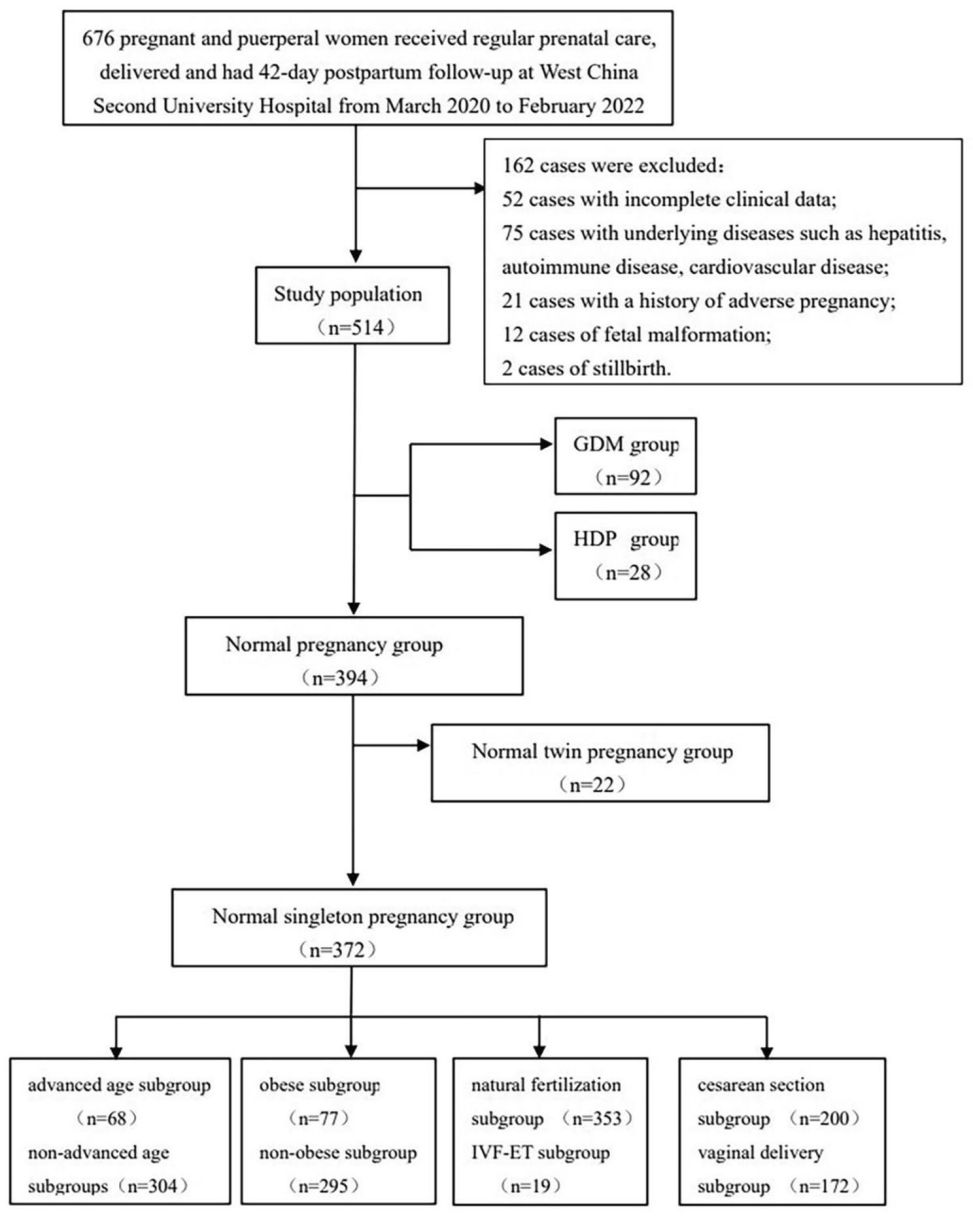
Flow chart of sample selection and grouping results of cohort 1

All participants were from southwestern China. Most of them were from Sichuan Province and Chongqing, and all of them were Han Chinese and had similar dietary habits. Cohort 1 included 514 pregnant and puerperal women who were divided into the normal pregnancy group (n = 394), the GDM group (n = 92) and the HDP group (n = 28). The HDP group included 8 participants with preeclampsia, and the HDP group and the GDM group all had singleton pregnancies. The normal pregnancy group was divided into the normal singleton pregnancy group (n = 372) and the normal twin pregnancy group (n = 22). The normal singleton pregnancy group was divided into four pairs of subgroups: advanced age (n = 68) and nonadvanced age (n = 304) subgroups, obese (n = 77) and nonobese (n = 295) subgroups, natural fertilization (n = 353) and IVF-ET (n = 19) subgroups, and caesarean section (n = 200) and vaginal delivery (172) subgroups. The detailed sample collection and grouping results of cohort 1 are shown in Fig. 1.

In cohort 1, no VTE occurred; there were only 11 participants who developed PPH due to uterine atony or a retained placenta and membranes, but no severe PPH occurred. The total labor duration of the whole vaginal delivery subgroup did not exceed 24 h, and no OHSS occurred in the IVF-ET subgroup. The basic clinical characteristics of participants in the normal singleton pregnancy group, the normal twin pregnancy group, the HDP group and the GDM group are shown in Table 1. The gestational week of delivery of the normal singleton pregnancy group was significantly greater than that of the other three groups (all P < 0.05). The caesarean delivery rate of the normal singleton pregnancy group was significantly lower than that of the other three groups (all P < 0.05). The other basic clinical characteristics of the normal singleton pregnancy group, including delivery age, pre-pregnancy BMI, the proportion of participants with more than 3 births and the conception mode, were not significantly different from those of the other three groups (all P > 0.05).

**Table 1:** The basic clinical characteristics of the participants in cohort 1

Maternal parameters	Normal singleton pregnancy group (n = 372)	Normal twin pregnancy group(n = 22)	HDP group (n = 28)	GDM group (n = 92)
Gestational week (w)	39.10 ± 1.70	35.91 ± 2.27*	37.45 ± 2.18*	38.73 ± 1.83*
Delivery age (y)				
< 35	30.26 ± 2.66	30.40 ± 2.64	30.44 ± 3.24	30.94 ± 2.54
≥ 35	37.15 ± 1.96	37.14 ± 2.41	36.33 ± 2.31	37.75 ± 2.19
Pre-pregnancy BMI (kg/m^2^)				
< 28	24.95 ± 1.83	24.49 ± 1.92	25.27 ± 1.97	24.73 ± 1.89
≥ 28	30.28 ± 2.21	31.59 ± 3.13	31.48 ± 2.49	30.12 ± 2.28
Parity(≥3)	3(0.76%)	1(4.55%)	1(3.57%)	3(3.26%)
Conception mode				
Natural fertilization	353(94.89%)	20(90.91%)	25(89.29%)	87(94.57%)
IVF-ET	19(5.11%)	2(9.09%)	3(10.71%)	5(5.43%)
Delivery mode				
Vaginal delivery	172(46.24%)	1(4.55%)*	1(3.57%)*	27(29.35%)*
Cesarean delivery	200(53.76%)	21(95.45%)*	27(96.43%)*	65(70.65%)*

Data are expressed as mean ± SD, or n (%)

Compared maternal parameters of the normal singleton pregnancy group with that of the other three groups

*, P-value < 0.05

In cohort 2, 29 women developed VTE within 24–48 h after caesarean section. All of them had different degrees of chest tightness, dyspnea, swelling and numbness of the lower extremities or sustained blood oxygen saturation of less than 95% under deoxygenation, and then compression ultrasound and computed tomography pulmonary angiography were performed. Finally, 28 cases of pulmonary embolism and 1 case of left calf intermuscular venous thrombosis were diagnosed. All of them were given individualized anticoagulation therapy according to the opinions of the vascular surgeon on duty, and the follow-up results indicated that they were successfully cured. Among the 29 women with VTE, 20 had normal singleton pregnancies. Table 2 shows the basic clinical characteristics of the 20 normal singleton pregnancies after caesarean section with VTE in cohort 2 and 200 normal singleton pregnancies after caesarean section without VTE (caesarean section subgroup) in cohort 1. There was no significant difference between the two groups in terms of the gestational week of delivery, delivery age, pre-pregnancy BMI, the proportion of participants with more than 3 prior births, conception mode and delivery mode (all P > 0.05).

**Table 2:** The basic clinical characteristics of the normal singleton pregnancies in cohort 2 and the cesarean section subgroup in cohort 1

Maternal parameters	Normal singleton pregnancies in cohort 2 (n = 20)	Cesarean section subgroup in cohort 1 (n = 200)	P
Gestational week (w)	38.71 ± 1.52	39.01 ± 1.33	0.262
Delivery age (y)			0.310
< 35	31.26 ± 2.51	30.78 ± 2.69	
≥ 35	37.56 ± 1.73	37.04 ± 2.11	
Pre-pregnancy BMI (kg/m^2^)			0.547
< 28	24.88 ± 1.87	24.35 ± 1.98	
≥ 28	31.35 ± 2.01	30.79 ± 2.67	
Parity(≥3)	0(0%)	1(0.5%)	0.892
Conception mode			0.064
Natural fertilization	19(95%)	194(97%)	
IVF-ET	1(5%)	6(3%)	

Data are expressed as mean ± SD, or n (%)

### Plasma D-D level and reference range of normal singleton pregnant and puerperal women

As shown in Table 3, the plasma D-D levels of 372 normal singleton pregnancies during the three trimesters and at 24 to 48 h postpartum and 42 days postpartum were 0.42 (0.30–0.57) mg/L, 1.04 (0.73–1.51) mg/L, 1.98 (1.31–2.82) mg/L, 1.88 (1.40–2.83) mg/L and 0.24 (0.13–0.36) mg/L, respectively. In the above five periods, the proportion of pregnant and puerperal women with plasma D-D levels > 0.5 mg/L accounted for 33.33%, 95.43%, 100%, 100%, and 13.44%, respectively. The correlation between gestational week and plasma D-D level was statistically significant (P < 0.001), and the plasma D-D value increased by about 0.06 (95% CI: 0.03–0.08) mg/L for each additional week of gestational age. The differences in the plasma D-D levels during the 5 time periods were statistically significant (F = 329.327, P < 0.001).

**Table 3:** Plasma D-D level and reference range of 372 normal singleton pregnant and puerperal women

Sample time	Plasma D-D (mg/L)		F	P		Women with plasma D-D levels > 0.5 mg/L: n (%)
	M (P25-P75)	P95				
First trimester	0.42(0.30–0.57)	1.01	329.33	< 0.001		124(33.33)
Second trimester	1.04(0.73–1.51) *	3.17				355(95.43)
Third trimester	1.98(1.31–2.82) *†	5.35				372(100)
24–48 h postpartum	1.88(1.40–2.83) *†	5.47				372(100)
42 days postpartum	0.24(0.13–0.36) *†‡§	0.66				50(13.44)

M, median; P25, P75, P95, the 25th, 75th, and 95th percentiles

The plasma D-D levels (mg/L) are expressed as median and interquartile

*, When compared the plasma D-D level in the first trimester with that in other periods, P < 0.05; †, When compared the plasma D-D level in the second trimester with that in other periods, P < 0.05; ‡, When compared the plasma D-D level in the third trimester with that in other periods, P < 0.05; §, When compared the plasma D-D level at 24–48 h postpartum with that in other periods, P < 0.05

In nonpregnant women, plasma D-D is mainly used to assist in the exclusion of a diagnosis of VTE, with a negative predictive value of nearly 100%. When the plasma D-D level exceeds the upper limit of the reference range it is considered abnormal, and the occurrence of VTE needs to be monitored. Therefore, a one-sided upper limit (the 95th percentile) was used as the threshold of the plasma D-D level. According to the formula and combined with clinical practice, at least 347 samples are needed to establish a reliable reference range. Our study included 372 normal singleton pregnant and parturient women. As shown in Table 3, the 95% reference range of plasma D-D in normal singleton pregnant women and parturient women was ≤ 1.01 mg/L in the first trimester, ≤ 3.17 mg/L in the second trimester, ≤ 5.35 mg/L in the third trimester, ≤ 5.47 mg/L at 24 to 48 h postpartum, and ≤ 0.66 mg/L at 42 days postpartum.

### Effects of pregnancy- and childbirth-related factors on plasma D-D levels

**Fig. 2:**
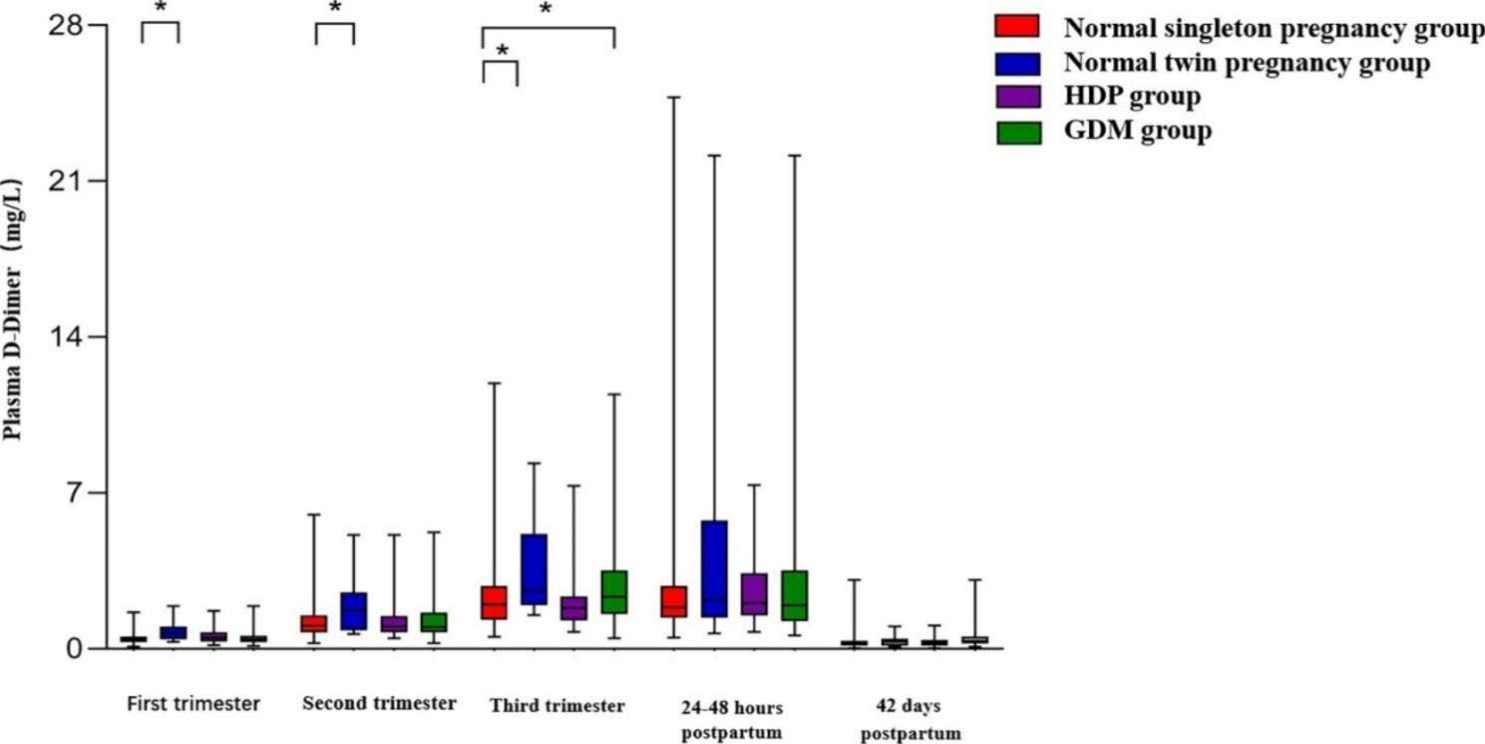
Plasma D-D levels in normal singleton pregnancy group, normal twin pregnancy group, HDP group and GDM group during pregnancy and puerperium. Compared the plasma D-D levels of the normal singleton pregnancy group with that of the other three groups. *, *P* < 0.05

As shown in Table 4; Fig. 2, the plasma D-D levels of the normal twin pregnancy group were significantly higher than those of the normal singleton pregnancy group during pregnancy (P < 0.05), and the plasma D-D levels showed no significant difference between the two groups during puerperium (P > 0.05). There was no significant difference in plasma D-D levels between the normal singleton pregnancy group and the HDP group during pregnancy and puerperium (P > 0.05). The plasma D-D levels of the GDM group in the third trimester were significantly higher than that of the normal singleton pregnancy group (P < 0.05), and the plasma D-D levels showed no significant difference between the two groups during the other 4 periods (P > 0.05).

**Table 4:** Comparison of plasma D-D levels between normal singleton pregnancy group and normal twin pregnancy group, HDP group and GDM group

Sample time	Normal singleton pregnancy group (n = 372)	Normal twin pregnancy group(n = 22)	HDP group (n = 28)	GDM group (n = 92)
First trimester	0.42(0.30–0.57)	0.70(0.44–0.98) *	0.52(0.33–0.76)	0.41(0.31–0.58)
Second trimester	1.04(0.73–1.51)	1.75(0.83–2.54) *	1.01(0.75–1.47)	0.31(0.74–1.63)
Third trimester	1.98(1.31–2.82)	2.61(1.96–5.15) *	1.84(1.26–2.36)	2.33(1.57–3.50) *
24–48 h postpartum	1.88(1.40–2.83)	2.20(1.40–5.77)	2.07(1.50–3.39)	1.97(0.25–3.53)
42 days postpartum	0.24(0.13–0.36)	0.34(0.23–0.57)	0.3(0.15–0.47)	0.27(0.14–0.40)

The plasma D-D levels (mg/L) are expressed as median and interquartile

Compared the plasma D-D levels of the normal singleton pregnancy group with that of the other three groups

*, P-value < 0.05

As shown in Table 5, the plasma D-D levels of the advanced age subgroup at 24–48 h postpartum were significantly higher than that of the nonadvanced age subgroup (P < 0.05), and there was no significant difference in plasma D-D levels between this pair of subgroups during the other four periods (all P > 0.05). There was no significant difference in plasma D-D levels between the obese and nonobese subgroups for all five periods (all P > 0.05), and no significant difference was evident in plasma D-D levels between the natural fertilization and IVF-ET subgroups during all five periods (all P > 0.05). The plasma D-D levels of the caesarean section subgroup at 24–48 h postpartum were significantly higher than that of the vaginal delivery subgroup (P < 0.05), and there was no significant difference in plasma D-D levels between this pair of subgroups at 42 days postpartum (P > 0.05).

**Table 5:** Comparison of plasma D-D levels between four pairs of subgroups

Sample time	Subgroups 1				Subgroups 2				Subgroups 3				Subgroups 4		
	Advanced age subgroup (n = 68)	Nonadvanced age subgroup (n = 304)	P		Obese subgroup (n = 77)	Nonobese subgroup (n = 295)	P		Natural fertilization subgroup (n = 353)	IVF-ET subgroup (n = 19)	P		Vaginal delivery subgroup (n = 172)	Cesarean section subgroup (n = 200)	P
First trimester	0.46 (0.31–0.67)	0.41 (0.30–0.55)	0.195		0.44 (0.33–0.64)	0.41 (0.30–0.56)	0.312		0.40 (0.30–0.56)	0.51 (0.42–0.70)	0.061		NA	NA	NA
Second trimester	1.08 (0.81–1.68)	1.04 (0.72–1.49)	0.365		1.11 (0.80–1.55)	0.99 (0.72–1.51)	0.318		1.03 (0.73–1.51)	1.10 (0.84–1.74)	0.451		NA	NA	NA
Third trimester	2.12 (1.41–2.81)	1.96 (1.29–2.84)	0.599		2.12 (1.41–2.97)	1.96 (1.27–2.80)	0.466		1.97 (1.31–2.82)	2.12 (1.50–2.98)	0.624		NA	NA	NA
24–48 h postpartum	1.96 (1.44–2.85)	1.67 (1.12–2.76)	0.043		1.81 (1.34–2.60)	1.92 (1.41–2.88)	0.229		1.92 (1.41–2.80)	1.64 (1.22–3.23)	0.605		1.75 (1.34–3.59)	2.11 (1.43–3.24)	0.005
42 days postpartum	0.24 (0.12–0.36)	0.24 (0.13–0.36)	0.675		0.26 (0.18–0.44)	0.23 (0.12–0.36)	0.059		0.24 (0.13–0.36)	0.26 (0.16–0.49)	0.380		0.23 (0.13–0.39)	0.24 (0.13–0.36)	0.634

The plasma D-D levels (mg/L) are expressed as median and interquartile

### Relationship between plasma D-D level and the occurrence of VTE during early puerperium after caesarean section

**Fig. 3:**
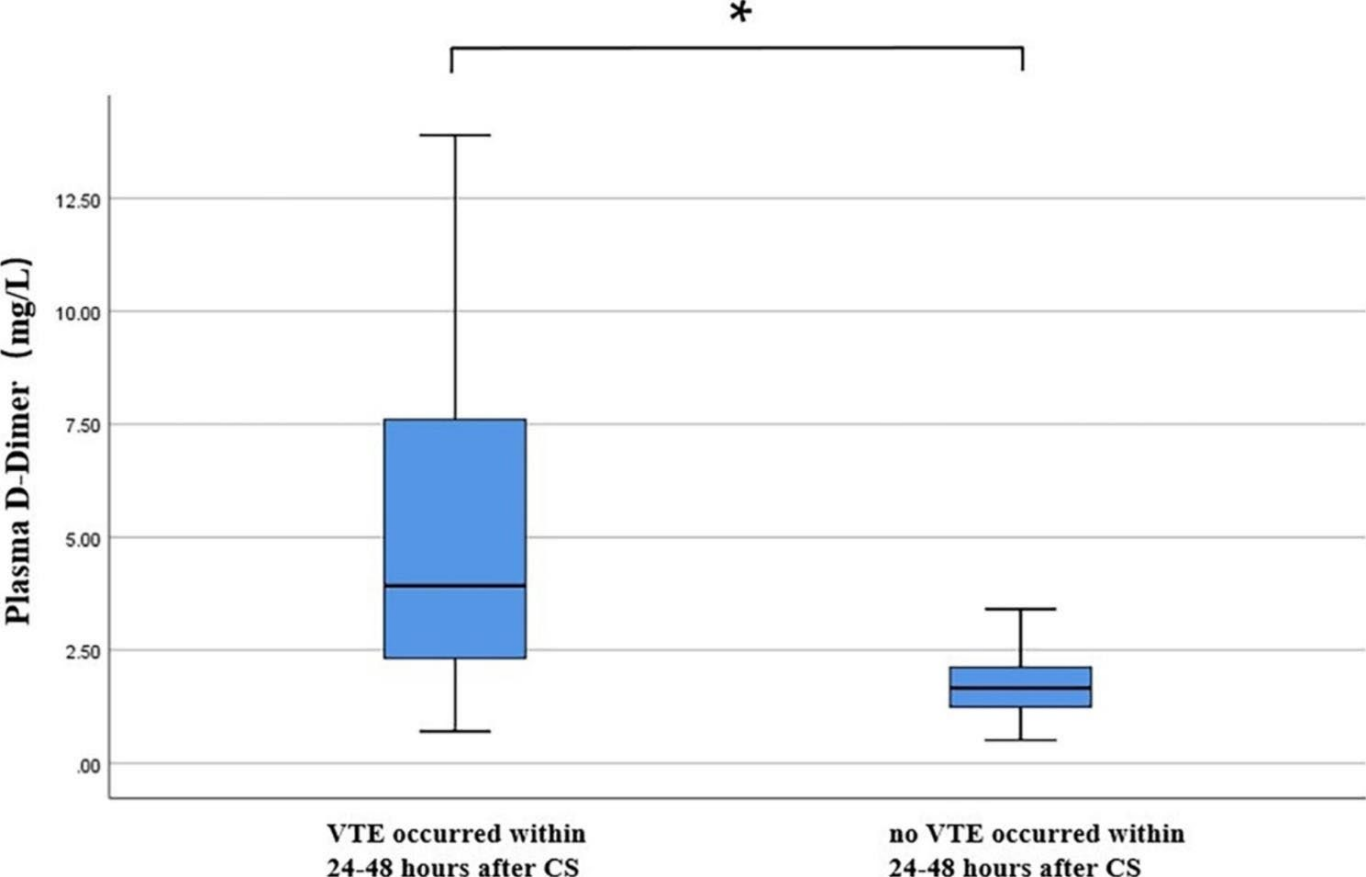
The plasma D-D levels of 20 normal singleton pregnant and puerperal women in cohort 2 and 200 women from the cesarean section subgroup in cohort 1. *, *P* = 0.033

**Fig. 4:**
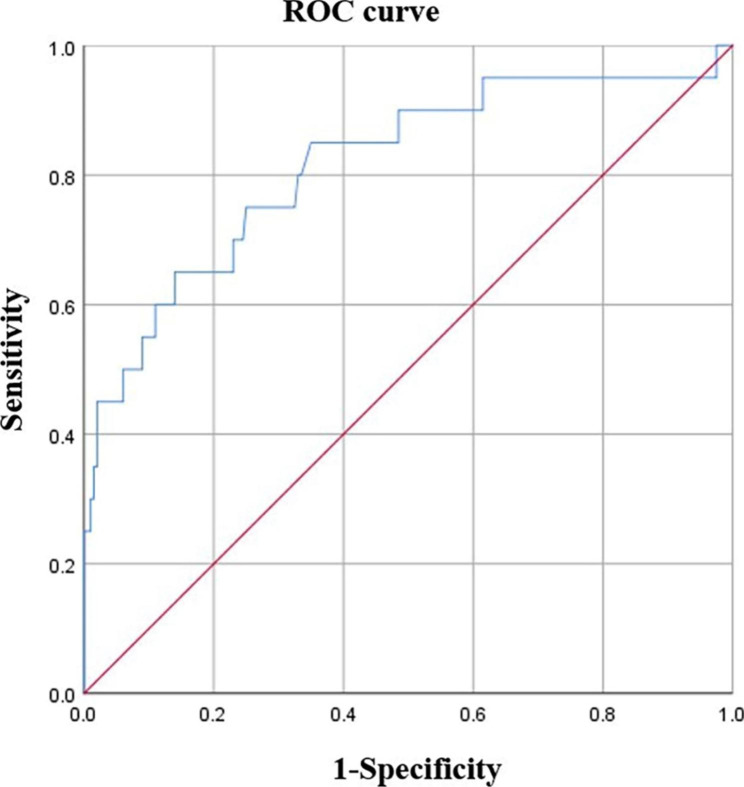
The sensitivity and specificity of plasma D-D screening for VTE occurred within 24–48 h after cesarean section. When the cut-off value of plasma D-D was 3.24 mg/L, the sensitivity, specificity and negative predictive value for exclusion diagnosing VTE were 65%, 86% and 96.1% respectively, and the area under the curve was 0.816, *P* < 0.001

As shown in Fig. 3, the plasma D-D levels of 20 normal singleton pregnant and puerperal women in cohort 2 who occurred VTE within 24–48 h after caesarean section were significantly higher than those of the caesarean section subgroup in cohort 1 [3.93 (2.23–7.91) mg/L vs. 2.11 (1.43–3.24) mg/L, P = 0.033]. The plasma D-D level at 24–48 h after caesarean section was associated with an increased risk of VTE (OR = 2.252, 95% CI: 1.611–3.149). As shown in Fig. 4, the ROC curve analysis found that the optimal cut-off value of plasma D-D for the diagnosis of exclusion of VTE during early puerperium after caesarean section was 3.24 mg/L, the sensitivity, specificity, and negative predictive value were 65%, 86% and 96.1%, respectively, and the area under the curve (AUC) was 0.816, P < 0.001.

## Discussion

Our study found that the correlation between gestational week and plasma D-D level was statistically significant, and the plasma D-D level increased with gestational week, which is roughly consistent with the findings of Murphy et al. and Jeremiah et al. (r = 0.70, P < 0.01; r = 0.36, P = 0.005) [22, 23], showing that with the progression of gestation, the physiological changes in the coagulation and fibrinolysis systems also progressed. The research findings of Mohmad et al. are consistent with those of the present study, and both concluded that plasma D-D levels peaked in the third trimester [24]. However, Murphy et al. found that the peak of plasma D-D appeared during early puerperium (within 24 h after delivery) [22]. Compared with the study of Murphy et al., the period of blood sample collection in the early puerperium (within 24–48 h after delivery) set in our study was longer. We speculate that the coagulation and fibrinolytic systems change rapidly after delivery, and it may be more useful to observe the complex changes in plasma D-D levels during pregnancy and puerperium if the frequency of blood sample collection could be increased during this period. In addition, our study found that plasma D-D levels tended to decrease gradually after delivery and basically returned to the nonpregnant level at 42 days postpartum, suggesting that the complex adaptive changes of the coagulation and fibrinolytic systems that occurred during pregnancy may gradually weaken with the termination of pregnancy and return to the normal nonpregnant coagulation and fibrinolysis status at 42 days postpartum. Consistent with the results of most existing studies [25–27], the plasma D-D thresholds during pregnancy and puerperium in our study were all higher than 0.5 mg/L, further indicating that the existing nonpregnant plasma D-D reference range is indeed not suitable for guiding the assessment of coagulation and fibrinolytic status of pregnant and puerperium women.

In the present study, the plasma D-D levels of the normal twin pregnancy group were significantly higher than those of the normal singleton pregnancy group during pregnancy, which is generally consistent with the results of two other studies [28, 29]. The plasma D-D levels of twin pregnancies were higher, which may be related to the larger placental volume and the greater secretion of progesterone, estrogen and fetal-related proteins in twin pregnancies, resulting in more significant changes in coagulation and fibrinolytic function. One study found lower platelet counts and antithrombin activity, and higher levels of fibrinogen and plasma D-D in twin pregnancies, suggesting greater blood hypercoagulation and fibrinolytic activity in women with twin pregnancies than in women with singleton pregnancies [30, 31]. In the postpartum period, such differences may therefore be eliminated due to the delivery of the fetuses and placentas. In the present study, the plasma D-D levels of the HDP group were not significantly different from those of the normal singleton pregnancy group during pregnancy and puerperium. Our research result is consistent with the findings of Higgins et al. [32]. However, due to systemic arteriolar spasm and damage to the vascular endothelium in HDP patients, the original dynamic balance between the coagulation and fibrinolytic systems is disrupted, especially in patients with preeclampsia. Preeclampsia is currently recognized as a risk factor for VTE, and the above effects are more pronounced, with blood often in a pathological hypercoagulable status [33, 34]. The majority of studies have concluded that maternal plasma D-D levels are significantly higher in those with HDP than in a normal singleton pregnancy and that plasma D-D levels may be associated with the severity of HDP and may even have predictive value for preeclampsia [35, 36]. Elevated plasma D-D level represents severe microthrombi formation, and severe microthrombi may lead to severe organ damage. The HDP group of our study received regular prenatal care throughout pregnancy, their blood pressures were under control, and no serious organ damage or adverse pregnancy outcomes happened. We speculate that these women may not have severe microthrombi formation, therefore the plasma D-D levels of them were not significantly higher. In addition, the correlation between HDP and plasma D-D levels might be better observed if the sample size of the HDP group could be expanded. In our study, the plasma D-D level of the GDM group was significantly higher than that of the normal singleton pregnancy group in the third trimester, which is consistent with the findings of Bellart et al. [37]. Due to the increase of sugar excretion and the fetus’ need for nutrients, the blood glucose levels of pregnant women decrease at the early stage of gestation. During the middle and advanced stage of pregnancy, the placenta secrets the progesterone, estrogen and human placental lactogen, which have the effect of antagonizing insulin, and the levels of these hormones reach the peak in late pregnancy, the status of relative hyposecretion of insulin causes the increase of blood glucose [38]. Long-term high blood glucose levels may lead to varying degrees of damage to the vascular endothelium, triggering the exogenous coagulation pathway and then leading to cascade activation of the coagulation system and secondary activation of the fibrinolytic system, and eventually to elevated plasma D-D levels. The levels of insulin-antagonizing hormones produced by the placenta decrease with the delivery of the placenta, and the original status of relative hyposecretion of insulin changes, and then the blood glucose gradually returns to the normal level. This may be the reason why the GDM group did not have higher plasma D-D levels during the peripartum period.

In the present study, the plasma D-D level of the advanced age subgroup was significantly higher than that of the nonadvanced age subgroup at 24–48 h postpartum. The results of Miyamoto et al. were generally consistent with our study [39], while some studies also found that the plasma D-D level of the advanced age subgroup was significantly higher in the third trimester [40]. In recent years, there has been a major shift in China’s family planning policy, with the successive introduction of two- and three-child policies, which has led to an increasing proportion of older pregnant and puerperal women. With increasing age, the elasticity of blood vessels decreases, and the vascular endothelium is damaged to a certain extent. The coagulation and fibrinolysis functions of older pregnant women may be affected to a certain extent, and the recovery of these functions is poor after delivery. Obesity is a risk factor for developing VTE during pregnancy and puerperium [41, 42]. Some studies have even shown that after age matching, compared with women with normal BMI, women with a higher BMI are more prone to develop VTE during pregnancy and puerperium [43]. Obese pregnant women may have disorders of lipid metabolism, decreased release of prostacyclin, and increased secretion of peroxidase, causing hemoconcentration and coagulation and fibrinolytic disorders. Therefore, we speculate that obese patients may have higher plasma D-D levels than nonobese patients, but an effect of obesity on maternal plasma D-D levels was not observed in our study. The small sample size of the obese subgroup may cause this result. There is another study also showing that BMI was not correlated with the plasma D-D level [44], which supports the conclusion of our study to a certain extent, but the results of that study are only based on nonpregnant women. There are few studies on the association between obesity and the plasma D-D level during pregnancy and puerperium, so it is necessary to explore the effect of obesity on maternal plasma D-D level in a large, multicenter maternal population in the future. A study including 18,000 pregnant women after IVF-ET found that the incidence of VTE during pregnancy was increased in the IVF-ET group compared with the spontaneous conception group (OR = 3, 95% CI: 2.1–4.3) [45]. However, our study did not find similar results. The risk of villous micro thrombosis increases after IVF-ET, which can lead to inadequate placental perfusion and local ischemic infarction, resulting in early abortion [46, 47]. Di Nisio et al. concluded that high plasma D-D levels in women after IVF-ET were associated with a higher risk of pregnancy failure [48]. This may be the reason for the current close monitoring of plasma D-D levels by reproductive medicine physicians in women after IVF-ET. All women after IVF-ET in our study had successfully conceived and had an uneventful pregnancy, implying that this group of women did not have the significantly high levels of plasma D-D seen in assisted reproductive conceptions complicated by OHSS, which explains the results that there was no significant difference in plasma D-D levels between the natural fertilization and IVF-ET subgroups during pregnancy and puerperium and IVF-ET subgroup didn’t have a higher incidence of VTE. And the effect of the small sample size on the results should also be considered.

Caesarean section is an independent risk factor for the development of VTE during puerperium, with a postoperative VTE prevalence of 0.03%, which is approximately four times higher than that of vaginal delivery [49]. Correspondingly, our study found that the plasma D-D level of the caesarean section subgroup at 24–48 h postpartum was significantly higher than that of the vaginal delivery subgroup, which is consistent with the results of many other studies [26, 50]. Damage to the venous blood vessel wall during caesarean section and alteration of the body’s internal environment by anesthetic drugs lead to activation of the maternal coagulation system and secondary activation of the fibrinolytic system, resulting in increased plasma D-D levels. The high level of plasma D-D during early puerperium after caesarean section corresponds to the high incidence of VTE, and all participants in cohort 2 of our study developed VTE in the early puerperium after caesarean section. Our study found that plasma D-D had a good value for the diagnosis of exclusion of VTE occurring within 24–48 h after caesarean section, and we set 3.24 mg/L as the optimal cut-off value for the plasma D-D level. However, this cut-off value is lower than the upper limit of the reference range of plasma D-D in the third trimester and at 24–48 h postpartum established in our study, which may be related to the small sample size of the population with VTE. In addition, the prospective cohort study of HU et al. included 12,451 older puerperal women and found that the optimal cut-off value of plasma D-D level for the exclusion of a diagnosis of VTE within 24 h after delivery was 5.545 mg/L [51]. This cut-off value is also higher than ours, probably because their study only focused on older puerperal women, and the period of blood sample collection during early puerperium was also different from our study. Unfortunately, since our study did not include a population with VTE during pregnancy, we could not further explore the correlation between plasma D-D levels and VTE occurring during pregnancy.

It should be noted that there were some differences in the baseline characteristics (including the gestational week of delivery and delivery mode) between the normal singleton pregnancy group and the normal twin pregnancy group, the HDP group, and the GDM group in our study. Due to the small sample size, the confounding factors could not be adjusted by the propensity score matching method and logistic regression stratified analysis, so the results of this study may have a certain bias. In the comparison between 20 normal singleton pregnant and puerperal women in cohort 2 and 200 women from the cesarean section subgroup in cohort 1, the sample sizes of the two groups differed considerably, there may be a bias in our results, and the sample size should be further expanded. Fortunately, there was no significant difference in baseline clinical characteristics between the two groups, which can minimize the bias caused by sample size differences to some extent. Our study is a prospective cohort study that enrolled the same study population throughout pregnancy and puerperium, reducing the impact of individual differences. The present study provides a continuous and dynamic changing trend of plasma D-D levels during the whole pregnancy and puerperium (including early puerperium and 42 days postpartum), with complete data and better reference values.

## Conclusion

This study may help to promote the rational application of plasma D-D on pregnant and puerperal women in the future and provide data reference for clinicians to use plasma D-D to dynamically observe the coagulation and fibrinolysis status of pregnant and puerperal women. A large-scale, multi-center, high-quality prospective study is still needed to clarify the pregnancy- and childbirth-related factors affecting plasma D-D levels, validate the reference range of plasma D-D suitable for pregnant and puerperal women, and explore the optimal cut-off value of plasma D-D for excluding a diagnosis of VTE during pregnancy and puerperium.

## Data Availability

The datasets analyzed during the current study are available from the corresponding author upon reasonable request.

## Abbreviations

VTE: Venous thromboembolism
D-D: D-dimer
CI: Confidence interval
OHSS: Ovarian hyperstimulation syndrome
GDM: Gestational diabetes mellitus
HDP: Hypertensive disorders of pregnancy
PPH: Postpartum hemorrhage
FIGO: International Federation of Gynecology and Obstetrics
AMA: Advanced maternal age
BMI: Body mass index
ISH: International Society of Hypertension
ADA: American Diabetes Association
IVF-ET: In vitro fertilization-embryo transplantation
ROC: Receiver operating characteristic
AUC: Area under the curve
